# Screening and Assessment of Antimicrobial Susceptibility of Periodontopathic Bacteria in Peruvian Patients with Periodontitis: A Pilot Study

**DOI:** 10.1155/2021/2695793

**Published:** 2021-02-24

**Authors:** Miguel Angel Aguilar-Luis, Leslie Casas Apayco, Carmen Tinco Valdez, María del Carmen De Lama-Odría, Claudia Weilg, Fernando Mazulis, Wilmer Gianfranco Silva-Caso, Juana Mercedes Del Valle-Mendoza

**Affiliations:** ^1^School of Medicine, Research and Innovation Center of the Health Sciences, Universidad Peruana de Ciencias Aplicadas, Lima, Peru; ^2^Laboratorio de Biología Molecular, Instituto de Investigación Nutricional, Lima, Peru; ^3^School of Dentistry, Universidad Peruana de Ciencias Aplicadas, Lima, Peru

## Abstract

**Background:**

Severe periodontal disease is highly prevalent worldwide, affecting 20% of the population between the ages of 35 and 44 years. The etiological epidemiology in Peru is scarce, even though some studies describe a prevalence of 48.5% of periodontal disease in the general population. Periodontitis is one of the most prevalent oral diseases associated with site-specific changes in the oral microbiota and it has been associated with a socioeconomic state. This study aimed to determine the etiology and resistance profile of bacteria identified in a group of Peruvian patients with periodontal disease.

**Methods:**

Six subgingival plaque samples were collected from eight patients with severe periodontitis. Bacterial identification was carried out by an initial culture, PCR amplification, and subsequently DNA sequencing. We evaluated the antibiotic susceptibility by the disk diffusion method.

**Results:**

Variable diversity in oral microbiota was identified in each one of the eight patients. The bacterial genus most frequently found was *Streptococcus* spp. (15/48, 31.3%) followed by *Rothia* spp. (11/48, 22.9%), *Actinomyces* spp. (9/48, 18.8%), and *Eikenella* spp. (4/48, 8.3%). The most common species found was *Rothia dentocariosa* (8/48, 16.7%). The antimicrobial susceptibility assay varied according to the species tested; however, among all the isolates evaluated, *Actinomyces naeslundii* was resistant to penicillin and tetracycline; *Eikenella corrodens* was resistant to dicloxacillin; and *Rothia dentocariosa* was resistant to amoxicillin + clavulanic acid and metronidazole but also susceptible to trimethoprim-sulfamethoxazole.

**Conclusions:**

The most prevalent periodontal bacterium found in this study was *Rothia dentocariosa.* Specific antimicrobial therapy is required to improve the treatment outcomes of patients with periodontal disease and avoid antibiotic resistance.

## 1. Introduction

Oral diseases remain highly prevalent affecting 3.9 billion people worldwide [1]. Oral diseases are the main cause of tooth loss. The most common infections in patients above and below 35 years are dental caries and periodontal disease, respectively. These conditions may explain the high prevalence of tooth loss in patients over the age of 60, reported to be as high as 25%. Worldwide, 15 to 20 percent of adults between 35 and 44 years old have severe periodontal disease and the prevalence increases in older patients [2]. In Peru, periodontal epidemiological data is scarce; however, Robello et al. described a prevalence of 48, 5% of periodontal disease in a study that included 1000 patients [3].

Periodontal disease is a common condition affecting the dental supporting structure. It is classified as gingivitis or periodontitis. Both are inflammatory processes but are distinguished by the presence of alveolar bone involvement in periodontitis [4]. The main predisposing risk factors for periodontal disease are subgingival plaque accumulation, stress, smoking, immunological disorders, nutritional deficiency, traumatic occlusion, and medical conditions. Periodontal disease represents a well-known risk factor for several systemic diseases. It is associated with an increased risk of coronary and cerebrovascular disease [5, 6]. Chronic periodontitis can have a negative effect on metabolic control in individuals with diabetes mellitus, as it contributes to increased inflammatory burden and enhanced insulin resistance [7, 8]. Also, odontogenic infections may disseminate hematogenously to prosthetic or native heart valves, joint replacements, or any other prosthetic devices, which is why antibiotic prophylaxis is essential prior to any invasive dental procedure. In pregnant women, periodontal disease has been associated with preterm birth and low birth weight. Although the available studies cannot provide conclusive evidence that these pregnancy complications resulted from periodontal disease [9], there is evidence that periodontal treatment significantly reduced the preterm and low birth weight rate in a population of pregnant women with a periodontal disease from Santiago de Chile [9]. Furthermore, periodontitis has been associated with smoking-related cancers in nonsmoking men [10].

Periodontitis covers the major plaque‐associated periodontal diseases. It could be classified as necrotizing periodontal disease, periodontitis as manifestation of systemic diseases, and periodontitis (stages and grades) [11]. Periodontal bacteria penetrate the gingival epithelium and elicit an inflammatory host response that ultimately results in damage to the supporting structures of the teeth [12]. Human dental plaque is composed of an extensive variety of microorganisms. In fact, up to 800 species have been identified so far [13]. It has been described that *Streptococcus*, *Peptostreptococcus*, *Veillonella*, and diphtheroid account for more than 80% of healthy oral cavity flora. Vengerfeldt et al. found that the microbial communities that colonize the root canal in antibiotic-naive patients with periodontal disease were *Firmicutes*, *Bacteroidetes*, *Actinobacteria*, *Fusobacteria*, *Proteobacteria*, *Spirochaetes*, *Tenericutes*, and *Synergistetes* [14].

Due to microbial specificity in periodontitis, the use of antibiotics along with surgical management is suitable in certain cases of severe periodontitis or periodontal disease in patients with medical risk factors [15]. Nevertheless, there is a slight question of whether certain specific microorganisms are related to specific stages of periodontal diseases [13]. In periodontitis, one of the clinical manifestations is the loss of connective tissue leading to deep pockets with Gram-negative and virulent strains of bacteria. In such cases, antimicrobials would prove to provide valuable assistance in cases where periodontal clinical treatment alone was not capable of effectively eliminating all periodontal pathogens [16]. The choice of specific antibiotics for the treatment of periodontal diseases is based on the available knowledge of human oral flora; however, there is an increasing tendency antimicrobial drug resistance rates worldwide [17].

The present study aimed to screen and assess the antimicrobial susceptibility of periodontopathic bacteria in Peruvian patients with periodontitis, a pilot study.

## 2. Materials and Methods

### 2.1. Patients Selection

Eight patients diagnosed with periodontitis were included in this study. Six periodontal pocket depths in the range from 4 mm to 8 mm were selected for each patient to collect the total sample. None of the patients received any antibiotic or periodontal treatment prior to sample collection. Also, they did not have any systemic disease and were nonsmoking.

### 2.2. Sample Collection

The subgingival plaque samples were collected from each patient, following the standard collection protocol as described by Jervoe-Storm et al. [18]. Before sterile endodontic paper points were used to collect the samples at pocket depths, supragingival drying was carried out to avoid cross-contamination. Samples were then transported in modified Dulbecco's Modified Eagle's Medium (DMEM, Sigma-Aldrich Inc, Missouri, United States) at 4°C for further use.

### 2.3. Ethics Statement

This study was approved by the Ethics Committee from Universidad Peruana de Ciencias Aplicadas in Lima, Peru.

### 2.4. Bacterial Isolation and Identification

A volume of 200 *µ*L of samples was cultured on Brucella Agar plates (Becton, Dickinson and Company, Maryland, United States), supplemented with 10% sheep blood, vitamin K (1 *µ*g/mL), hemin (5 *µ*g/mL), and kanamycin (50 *µ*g/mL) and incubated at 37°C under anaerobic conditions. The plates were reviewed every 24 hours until bacterial growth was observed.

Specific colonies were selected according to morphological characteristics. PCR was performed by amplifying a universal 16S ribosomal sequence on the studied microorganisms (27F 5'-AGAGTTTGATCCTGGCTCAG-3'; 1492R 5'-GGTTACCTTGTTACGACTT-3'), as previously described [19]. The amplified products obtained were sequenced using an ABI-3730XL DNA sequencer by Macrogen Inc. (Seoul, Korea) and DNA sequences were compared with published sequences retrieved from the GenBank database (National Center for Biotechnology Information, Bethesda, MD, Unites states).

### 2.5. Bacterial Susceptibility

Bacterial susceptibility was evaluated using the disk diffusion method. The following antimicrobials obtained commercially were evaluated (Liofilchem® s.r.l., Roseto degli Abruzzi, Italy): penicillin 10 U (P), ciprofloxacin 5 *µ*g (CIP), azithromycin 15 *µ*g (AZM), clindamycin 2 *µ*g (CD), tetracycline 30 *µ*g (TE), dicloxacillin 1 *µ*g (DCX), ampicillin 10 *µ*g (AMP), trimethoprim-sulfamethoxazole 25 *µ*g (SXT), amoxicillin 10 *µ*g (AML), amoxicillin + clavulanic acid 30 *µ*g (20/10) (AUG), and metronidazole 5 *µ*g (MTZ). Susceptibility breakpoints were interpreted according to Clinical and Laboratory Standards Institute (CLSI) breakpoint criteria. Due to the lack of CSLI criteria in some species in this study, such as *Rothia* spp. and *E. corrodens*, the categorical interpretations (susceptible, intermediate, or resistant) were carried out following the criteria for *Staphylococcus* spp. [20]. European Committee on Antimicrobial Susceptibility Testing (EUCAST) breakpoints was followed when the CLSI antimicrobial breakpoints could not be established [21].

## 3. Results

### 3.1. Bacterial Isolation

A total of 6 samples were collected from each of the 8 patients with periodontitis. Six patients (75%) were female, and 2 (25%) were male. The average age of the study population was 51.13 years.

**Figure 1:**
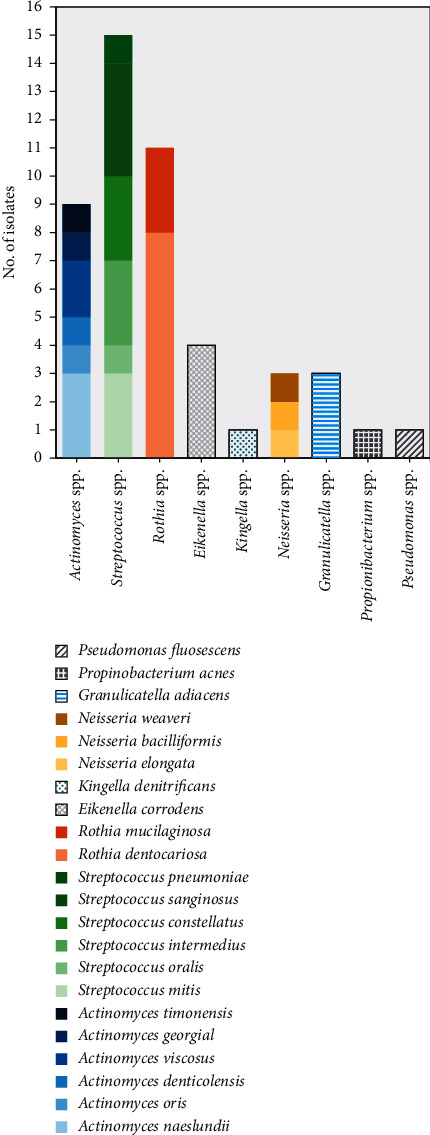
Frequency of identified samples among patients with chronic severe periodontitis.

A high diversity of microbes was found among all patients. At least 3 different bacterial genera were identified in the periodontal samples of each patient and the highest diversity of microorganisms was found in 3 (37.5%) patients with the identification of at least 5 different genera. The most frequent bacterial genus identified in all the samples was *Streptococcus* spp. (15/48, 31.3%), followed by *Rothia* spp. (10/48, 22.3%), *Actinomyces* spp. (9/48, 18.8%), and *Eikenella* spp. (4/48, 8.3%) (Table 1). In the study population, *Rothia dentocariosa* was identified in all the patient samples tested and was also the most frequently identified species in the population with 8/48 isolates (16.7%). In contrast, *Eikenella corrodens* was found in half of all patients tested (4 isolates) and *Actinomyces naeslundii* along with *Granulicatella adiacens* in 3 different patients (3 isolates) (Figure 1).

### 3.2. Antibacterial Resistance

Based on the frequency of identification within the study group, we selected 4 different microorganisms to determine their antimicrobial susceptibility: *Rothia dentocariosa*, *Eikenella corrodens*, *Granulicatella adiacens*, and *Actinomyces naeslundii*. The study found that the antimicrobial susceptibility patterns were very variable according to each microorganism. *Rothia dentocariosa* showed resistance to metronidazole (MTZ), amoxicillin + clavulanic acid (AUG), and amoxicillin (AML). Meanwhile, *Eikenella corrodens* was resistant to ciprofloxacin (CIP), azithromycin (AZM), clindamycin (CD), trimethoprim-sulfamethoxazole (SXT), dicloxacillin (DCX), and ampicillin (AMP). *Granulicatella adiacens* was resistant to ciprofloxacin (CIP), trimethoprim-sulfamethoxazole (SXT), dicloxacillin (DCX), ampicillin (AMP), and metronidazole (MTZ). Finally, *Actinomyces naeslundii* exhibited resistance to 7 of the 11 antibiotics tested and was susceptible to ciprofloxacin (CIP), dicloxacillin (DCX), amoxicillin + clavulanic acid (AUG), and amoxicillin (AML) (Table 1). In the antimicrobial resistance test, it was found that *Actinomyces naeslundii* was resistant to penicillin (P) and tetracycline (TE) while other bacteria were susceptible to those antibiotics; only *Eikenella corrodens* was resistant to dicloxacillin (DCX); and only *Rothia dentocariosa* was resistant to amoxicillin + clavulanic acid (AUG) and metronidazole (MTZ) but also susceptible to trimethoprim-sulfamethoxazole (SXT).

## 4. Discussion

Periodontal disease is a diagnosable, treatable, and potentially preventable condition. The prevalence of periodontal disease in Peru is nearly 50% [3] and it could be estimated that this number is higher in rural areas where inadequate oral hygiene is more common. Several other risk factors have been established, such as increasing age and hormonal effects, with the exacerbation of the disease during puberty, menstruation, and pregnancy [6, 9, 10]. Furthermore, certain underlying diseases may result in a predisposition to periodontal disease, such as rheumatoid arthritis, genetic disorders that impair neutrophil function, and diabetes mellitus. On the other hand, periodontal disease may be a risk factor for a number of systematic diseases [7, 8, 22].

The evidence in the literature that supports the etiological role of bacteria in periodontal diseases is overwhelming. Subgingival plaque is a complex microbial ecosystem with more than 800 microbial species of microorganisms. Some of these microbial species have been identified as the causative organisms for periodontal disease under the effect of local and systemic causes [8, 13, 15]. Among the oral flora, the most important are *Porphyromonas gingivalis* (seen in chronic periodontitis), *Aggregatibacter actinomycetemcomitans* (seen in aggressive periodontitis), *Bacteroides* sp., *Treponema* sp., *Fusobacterium* sp., *Prevotella* sp., *Campylobacter* sp., and *Eikenella* [23, 24]. The Socransky complexes, red and orange complex microorganisms, were commonly seen in 5 mm and deeper and attachment loss cases. The first or red complex was composed of *Bacteroides forsythus*, *Porphyromonas gingivalis*, and *Treponema denticola*. This complex was strongly associated with bleeding on probing; hence, it was categorized as being more closely associated with severe forms of periodontal disease. The second more related complex was labeled with the color orange. It included members of the *Fusobacterium nucleatum/periodonticum* subspecies, *Prevotella nigrescens*, *Prevotella intermedia*, and *Peptostreptococcus*. The orange complex was associated with the following species: *Eubacterium nodatum*, *Campylobacter rectus*, *Campylobacter showae*, *Streptococcus constellatus*, and *Campylobacter gracilis*. The yellow complex was comprised of *Streptococcus sanguinis*, *Streptococcus oralis*, *Streptococcus mitis*, *Streptococcus gordonii*, and *Streptococcus intermedius*. The green complex consisted of *Eikenella corrodens*, *Campylobacter concisus*, *Actinobacillus actinomycetemcomitans* serotype A, and three *Capnocytophaga* species. The A-A complex included *Actinomyces naeslundii*, *Actinomyces odontolyticus*, *Actinobacillus actinomycetemcomitans* serotype B, and *Veillonella parvula* (purple complex) [25].

The most frequently isolated genus in this study was *Streptococcus* spp., which belongs to the yellow complex and also *Rothia* spp. (22.9% of the samples). It should be mentioned that Rothia species have previously been described as part of the oral cavity flora in healthy patients [26]. *Actinomyces naeslundii* was primarily associated with oral plaque but may also be a factor in periodontal disease and opportunistic infections when invading tissues (18.8% of samples), which is part of the A-A complex. The absence of the most common periodontopathogens (*Aggregatibacter actinomycetemcomitans*, *Fusobacterium nucleatum*, *Peptostreptococcus micros*, *Porphyromonas gingivalis*, *Prevotella intermedia*, *Treponema denticola*, and *Treponema forsythia*) could reflect the presence of periodontal disease in the initial stages [23,24].

This study also found positive samples for microorganisms related to periodontitis disease, such as *Eikenella corrodens*, *Granulicatella adiacens* and *Neisseria* spp., but in a lesser proportion (Figure 1). *Eikenella corrodens* is a pleomorphic, Gram-negative bacteria, HACEK member. It belongs to the green complex of bacteria associated with periodontitis, but it has also been isolated in patients with infective endocarditis and liver abscess [26].

A large array of antibiotics is used as adjuncts to nonsurgical and surgical therapy in the treatment of periodontal disease. The commonly used antimicrobials in periodontal therapy are tetracycline, metronidazole, penicillin, macrolides, ciprofloxacin, and clindamycin. Metronidazole and amoxicillin are reported to be the most commonly used combination antibiotic regimen [27].

In this study, the antimicrobial susceptibility of the periodontopathogenic bacteria was studied for each microorganism. *Eikenella corrodens* has previously been described as resistant to clindamycin and metronidazole [26,28], which corresponds with the findings in this study. The isolated *Eikenella corrodens* was also resistant to trimethoprim-sulfamethoxazole (SXT) but susceptible to penicillin (P) (Table 1). *Actinomyces naeslundii* is a Gram-positive anaerobic bacillus. The isolated *Actinomyces naeslundii* in our study was susceptible amoxicillin (AML), but also to ciprofloxacin (CIP), dicloxacillin (DCX), and amoxicillin + clavulanic acid (AUG), and it was resistant to other antibiotics assessed. In contrast, *Actinomyces* spp. are susceptible only to penicillin and amoxicillin [29] (Table 1). Furthermore, *Granulicatella adiacens* is a Gram-positive coccus belonging to the family of “nutritional variant streptococci” (NVS). Infective endocarditis due to NVS is rare but is associated with significant mortality and morbidity [30]. *Granulicatella adiacens* has also been related to septic arthritis and bacterial abscesses [31]. Antimicrobial susceptibility tests on NVS report sensitivity to B-lactam antibiotics [32]. The isolated *Granulicatella adiacens* was susceptible to penicillin (P); however, it was resistant to ampicillin (AMP) (Table 1). In relation to *Rothia dentocariosa*, it is a pleomorphic, Gram-positive bacteria that commonly colonizes the mouth and throat and it rarely causes infective endocarditis, mainly in patients with previous heart conditions [26,33]. The isolated *Rothia dentocariosa* was resistant to metronidazole (MTZ), amoxicillin + clavulanic acid (AUG), and amoxicillin (AML) (Table 1).

The antimicrobial susceptibility tests in this study showed that the isolated microorganisms were susceptible to penicillin (P), except for *Actinomyces naeslundii.* However, most of the bacteria were also resistant to clindamycin (CD) and trimethoprim-sulfamethoxazole (SXT), which are commonly used antibiotics in dentistry. Biofilms have significantly high antimicrobial resistance when compared with their free-floating counterparts, which leads to severe concerns in the treatment procedure. Antimicrobial resistance of microorganisms is inherent or arises because of the emergence of resistant strains of bacteria [27, 34]. A few studies have evaluated the increase in antimicrobial resistance in oral biofilm postantimicrobial therapy. Feres et al. have reported patients with chronic periodontitis treated by nonsurgical periodontal treatment followed by oral antimicrobial therapy to have antibiotic-resistant species in their saliva and plaque samples [35]. A significant reduction of red and orange complex species in patients that received azithromycin or metronidazole along with surgical management has been seen [34].

The results showed that it is an important specific antimicrobial therapy as part of successful periodontitis treatment that also avoids an increase in antibiotic resistance of periodontopathic bacteria responsible for infectious complications, such as endocarditis or other complications associated with oral-hematogenous dissemination. It is relevant to highlight the significance of microbial resistance in antibiotics, thereby meaning it should be used more responsibly to prevent the spread of resistant microbial strains [17,35]. Further antimicrobial resistance studies are needed to determine a better antimicrobial therapy for periodontal diseases in the Peruvian population.

## 5. Conclusion

Bacterial species of medical importance were detected in patients with periodontitis, especially *Rothia dentocariosa*, which was most prevalent in this study. *Actinomyces naeslundii* was resistant to most of the antibiotics evaluated.

## Figures and Tables

**Table 1:** Antimicrobial susceptibility of identified periodontal bacteria by disk diffusion tests.

Isolated strains	Disk diffusion tests: antibiotics										
	P	CIP	AZM	CD	TE	SXT	DCX	AMP	MTZ	AUG	AML
*Rothia dentocariosa*	S	S	S	I	S	S	S	S	R	R	R
*Eikenella corrodens*	S	R	R	R	S	R	R	R	S	S	S
*Granulicatella adiacens*	S	R	S	I	S	R	S	R	R	S	S
*Actinomyces naeslundii*	R	S	R	R	R	R	S	R	R	S	S

Inhibition zone values: susceptible (S), intermediate (I), and resistant (R). “I” indicates that the result should be considered equivocal. Disk diffusion tests were repeated for each strain 2 times. Antibiotics: penicillin (P), ciprofloxacin (CIP), azithromycin (AZM), clindamycin (CD), tetracycline (TE), dicloxacillin (DCX), ampicillin (AMP), trimethoprim-sulfamethoxazole (SXT), amoxicillin (AML), amoxicillin + clavulanic acid (AUG), and metronidazole (MTZ).

## Data Availability

Abstraction format used in the study and dataset are available and accessible from the corresponding author upon request https://figshare.com/articles/dataset/Dataset_periodontitis_y2021m02/14072102.
